# Effects of whole blood storage in a polyolefin blood bag on platelets for acute normovolemic hemodilution

**DOI:** 10.1038/s41598-021-91725-y

**Published:** 2021-06-09

**Authors:** Yutaka Murata, Eriko Kusudo, Shuji Kawamoto, Kazuhiko Fukuda

**Affiliations:** Department of Anesthesia, Kyoto University Hospital, 54 Shogoin Kawahara-cho, Sakyo-ku, Kyoto, 606-8507 Japan

**Keywords:** Cardiovascular biology, Circulation, Biophysical methods

## Abstract

Acute normovolemic hemodilution (ANH) is a potential transfusion method for platelets, as well as for red blood cells. However, previous studies have shown that whole blood storage in ANH decreases platelet aggregability by 14.7–76.3% and that this decrease is not recovered by reinfusion. We investigated whether a new whole blood storage method for 6 h using a polyolefin bag, based on the platelet concentrates storage method, would maintain platelet function better than the conventional method using a polyvinyl chloride bag. We demonstrated that storage of whole blood in a polyolefin bag maintained ADP-induced aggregation rates at more than twofold higher than those in a polyvinyl chloride bag, and also significantly suppressed P-selectin expression, a platelet activation marker (ADP-induced aggregation rates: 24.6 ± 5.1% vs. 51.7 ± 11.5%, p = 0.002; P-selectin expression; 50.3 ± 8.4MFI vs. 31.6 ± 9.3MFI, p = 0.018). These results could be attributed to the high gas permeability of polyolefin, which lowered PCO_2_ and maintained a high pH with or without agitation. There were no significant changes in platelet count and red blood cell parameters due to the storage methods. Our results suggest that ANH using polyolefin bags is advantageous in improving hemostatic function compared to the conventional method.

## Introduction

Allogeneic blood transfusion is a common treatment for perioperative hemorrhage. Cardiac surgeries use large amounts of blood products, which consume 10–15% of the U.S. blood supply^1^. However, even small amounts of allogeneic blood transfusion increase mortality and serious complications^2^. For this reason and the global shortage of blood products^3,4^, use of allogeneic transfusion needs to be reduced.

**Figure 1:**
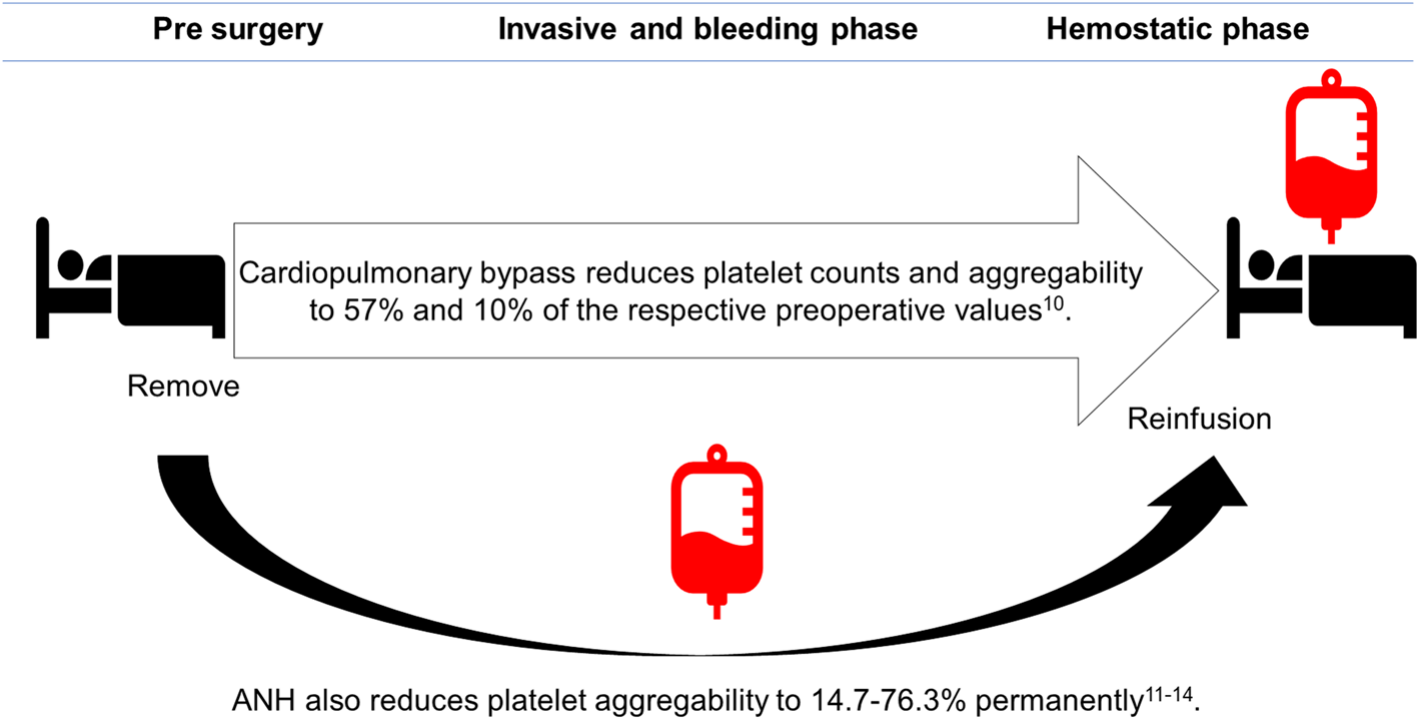
The conventional method of ANH cannot maintain platelet function. Cardiopulmonary bypass reduces platelet counts and function decreases to 57% and 10% of the respective preoperative values^10^. ANH also markedly reduces platelet function and these changes do not recover after transfusion^11–14^. This figure was drawn using Microsoft PowerPoint 2016 (Ver. 2103, https://www.microsoft.com/ja-jp/microsoft-365/powerpoint).

Acute normovolemic hemodilution (ANH) is a simple and low-cost transfusion method that may be an alternative option to allogeneic transfusion^1^. In ANH, 400–1200 mL of whole blood is removed from the patient immediately prior to an operation. The removed blood is mixed with CPDA solution and is stored without agitation at room temperature at the bedside. The circulating blood of the patient is diluted by crystalloid or colloid solutions, which reduces blood loss during surgery. At the conclusion of the operation, the stored autologous blood is restored to the patient (Fig. 1). ANH is safer than allogeneic blood transfusion because it does not cause graft-versus-host disease, infection or transfusion-related acute lung injury^1^. ANH has been covered by a national health insurance in Japan since 2016. Previous reports have shown that ANH substantially reduces intraoperative blood loss and allogeneic blood transfusion^1,5,6^.

ANH also reduces platelet and plasma transfusion significantly in cardiac surgeries^7^. Platelets of patients during cardiac surgeries are activated and consumed by cardiopulmonary bypass (CPB)^8^ through mechanisms of bypass- and heparin-induced activation, lack of extrinsic stimulating factors, and exposure to hypothermia during bypass^9^. Kotake et al.^10^ showed that post-CPB platelet counts and ADP-induced aggregability decrease to 57% and 10% of the respective preoperative values. ANH avoids potential platelet damage in CPB and can provide "fresh" platelets^8^. If ANH provides platelets with adequate hemostatic function, it can contribute to reduction in blood loss and allogeneic transfusion volume after CPB. However, several studies have found that the conventional method of ANH severely impairs platelet aggregability assessed by whole blood aggregometry, multiple electrode aggregometry, and impedance aggregometry (14.7–76.3% compared to pre-surgery) and that the function does not recover after transfusion^11–14^ (Fig. 1). Therefore, there is a need to develop a new ANH method with a high hemostatic effect by maintaining high platelet function in surgeries, including cardiac surgeries, in which massive bleeding can occur.

The storage conditions and expiration date of each blood production are shown in Table 1^1,15–23^. The major differences in storage for ANH and platelet concentrates are the bag material and use of agitation. Few studies mention the material of the storage bags used for ANH, but polyvinyl chloride (PVC) storage bags for red blood cells are generally used because of their excellent durability despite their low gas permeability^1,8,13,16,19,21^. In contrast, platelet concentrates are stored with agitation in bags made of highly oxygen-permeable materials such as polyolefin^16,22^, which provide sufficient oxygen for platelets to maintain aerobic respiration and ensure gas exchange between the storage medium and the atmosphere^24^.

**Table 1:** Blood storage conditions and expiration dates.

	Allogenic RBC	PABD	ANH	Platelet concentrates
Temperature	2–6 °C	2–6 °C	Room temperature	20–24 °C
Container	PVC bag	PVC bag	PVC bag	Polyolefin bag
Agitation	At rest	At rest	At rest	Agitation (60 rpm)
Solution	CPD∙MAP	CPDA	CPDA	ACD-A
Expiration date	21 days	35 days	Several hours	4 days

Blood storage conditions and expiration dates vary by country and facility. This table refers to some guidelines and our practice^1,15–23^.

*RBC* red blood cell, *PABD* preoperative autologous blood donation, *CPD-MAP* citrate–phosphate–dextrose∙mannitol–adenine–phosphate, *CPDA* citrate–phosphate–dextrose–adenine, *ACD-A* acid-citrate-dextrose formula A.

We hypothesized that use of a PVC bag at rest is responsible for the significant reduction in platelet function in conventional ANH. Therefore, we examined if whole blood storage in a polyolefin bag with or without agitation could maintain platelet function more effectively than that achieved by the conventional method.

## Results

**Figure 2:**
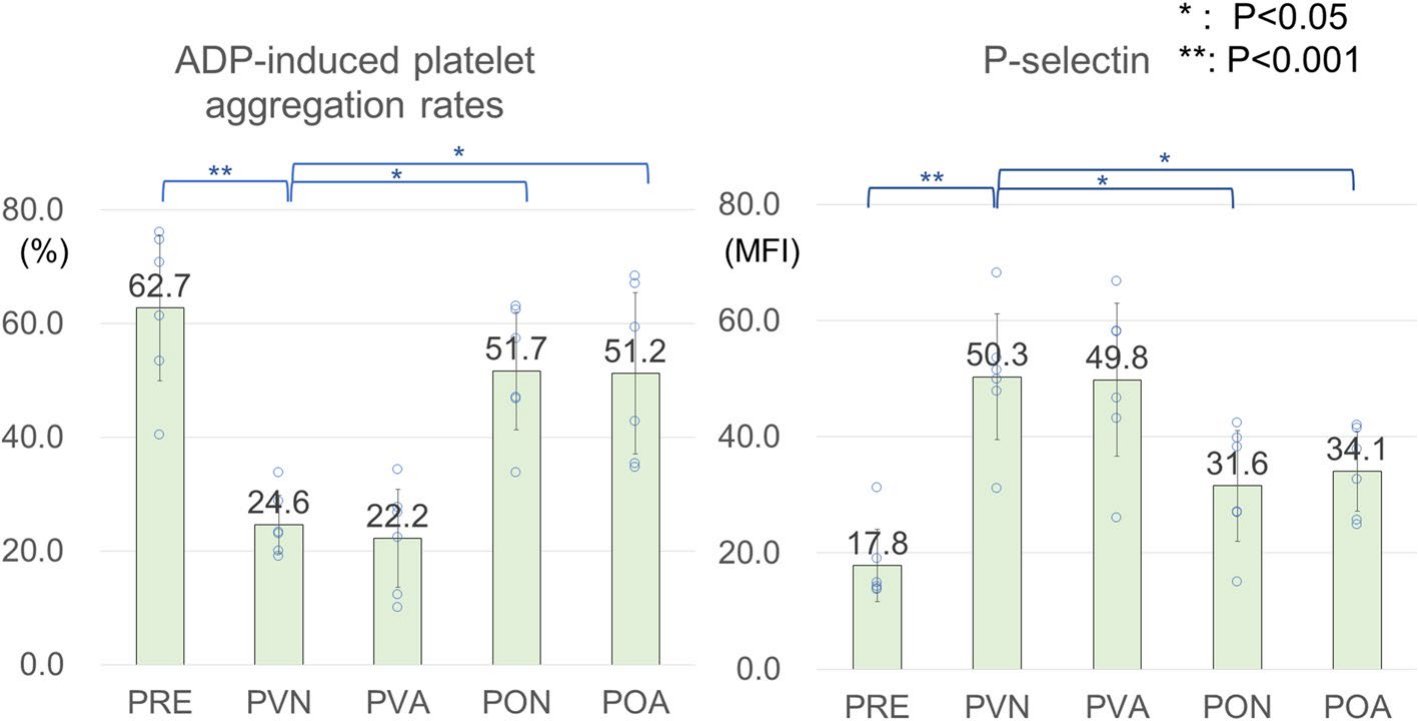
Comparison of ADP-induced platelet aggregation rates and P-selectin results. Compared to PRE, ADP-induced platelet aggregation rates in PVN and PVA storage decreased significantly and P-selectin expression of PVN and PVA increased significantly. Whole blood storage in polyolefin bags (PON and POA) maintained twofold higher ADP-induced platelet aggregation rates and a significantly lower P-selectin expression compared to PVN. *P < 0.05, **P < 0.001 vs. PVN by one-way ANOVA with a Dunnett multiple comparison test.

The results are shown in Table 2 and Fig. 2 (see "Methods" for the storage groups). First, the pH of venous blood mixed with CPDA before 6 h storage was lower than the normal venous blood pH (7.31–7.41)^25^ (Table 2 PRE). Compared to PRE, pH and ADP-induced platelet aggregation rates in PVN storage decreased significantly, and lactate and P-selectin increased significantly (Table 2 and Fig. 2 PRE–PVN). These results show that whole blood stored in the conventional ANH method severely impairs platelet function, as previous studies have shown^11–14^ (Fig. 1). In contrast, whole blood storage in polyolefin bags maintained significantly higher pH and ADP-induced platelet aggregation rates and significantly lower PCO_2_ and P-selectin, compared to those in the PVN group (Table 2 and Fig. 2 PON–PVN). PO_2_ in the PON group was relatively high, but not significantly higher than in the PVN group. No parameters in the PVA group differed significantly from those in the PVN group, and the POA and PON group showed significant differences from PVN in the same parameters. There were no significant changes in platelet count, mean platelet volume, and red blood cell parameters among all groups.

**Table 2:** Results for complete blood count and blood gas analysis in different storage conditions.

Item	PRE	PVN	PVA	PON	POA	P value (*P < 0.05)			
						PRE–PVN	PVA–PVN	PON–PVN	POA–PVN
Platelet count (10^4^/μL)	17.1 (2.7)	17.4 (3.2)	16.8 (3.5)	16.9 (3.1)	17.4 (2.5)	1.000	0.994	0.998	1.000
Hemoglobin (g/dL)	13.0 (1.2)	12.8 (1.2)	12.1 (0.8)	13.4 (1.0)	13.1 (1.1)	0.995	0.689	0.706	0.976
Hematocrit (%)	37.6 (3.2)	36.6 (3.1)	34.5 (2.9)	38.7 (2.5)	37.5 (3.5)	0.965	0.603	0.652	0.973
Mean corpuscular volume (fL)	88.5 (1.6)	88.6 (1.5)	88.3 (1.8)	88.5 (1.5)	88.3 (1.4)	1.000	0.997	1.000	0.994
Mean platelet volume (fL)	8.0 (0.3)	8.1 (0.3)	8.1 (0.3)	8.2 (0.2)	8.2 (0.3)	0.828	1.000	0.918	0.918
pH	7.038 (0.102)	6.919 (0.035)	6.912 (0.071)	7.053 (0.063)	7.029 (0.024)	0.011*	0.999	0.004*	0.020*
PCO_2_ (mmHg)	71.2 (13.0)	81.3 (11.0)	81.6 (11.6)	64.2 (1.5)	63.6 (6.5)	0.348*	1.000	0.045*	0.037*
PO_2_ (mmHg)	46.1 (23.9)	49.7 (23.2)	54.3 (26.0)	62.4 (35.0)	64.3 (35.7)	0.999	0.997	0.901	0.850
HCO_3_act (mM)	18.7 (3.4)	16.2 (1.7)	15.8 (1.8)	17.6 (2.5)	16.4 (1.9)	0.260	0.994	0.756	1.000
Lactate (mg/dL)	14.1 (8.2)	24.3 (5.7)	23.5 (5.0)	26.0 (6.0)	25.2 (5.1)	0.024*	0.998	0.965	0.997

Data are expressed as mean (SD). N = 6.

*P < 0.05 vs. PVN by one-way ANOVA with a Dunnett multiple comparison test.

## Discussion

In this study, we showed that whole blood storage in a polyolefin bag maintained higher ADP-induced platelet aggregation rates and lower P-selectin expression, compared to the conventional method used in ANH (Table 2 and Fig. 2). The decrease in aggregation rate measured by light transmittance aggregometry correlates with the amount of bleeding^26,27^. P-selectin (CD62P) is a membrane protein present in α granules of platelets, and its expression level on the unstimulated platelet surface is a common measure of platelet activations^28^. Platelets stored in the container are affected by various factors including duration of storage, temperature, pH, solution, gas permeability of the container for O_2_ and CO_2_ and interruption of agitation^29^. The platelet injuries associated with extracorporeal storage are called platelet storage lesions (PSLs)^30^, which lead to platelet activation via the necrotic and apoptotic processes, resulting the reduction of survival in vivo and hemostatic activity after transfusion^29^. Thus, decline of agonist-induced aggregation rates, P-selectin expression, morphological change and increased lactate level are the major manifestations of PSLs. ANH using polyolefin bags is advantageous in reducing PSLs and improving hemostatic function over the conventional method.

Some studies have shown that platelet function of whole blood stored in blood bags markedly decreases and does not recover after transfusion in ANH. Ramnarine et al. showed that collagen-induced formation of large stable hemostatic aggregates (platelet macroaggregation) were markedly decreased to 29.4% after collecting blood into a CPDA bag and to 14.7% after 80 min of storage, compared to that before the collection, and that this deterioration was largely irreversible by the reinfusion of ANH^12^. Scott et al. showed that blood in bag storage with CPDA for 300 min had significantly reduced platelet function, as measured by thrombin receptor activating peptide stimulation in multiple electrode aggregometry (Multiplate) analysis and maximum clot formation on ROTEM EXTEM^13^. Gallandat Huet et al. showed platelet aggregation response measured by Multiplate in ex-vivo stored blood with heparin decreased compared to the pre-CPB levels^11^. Therefore, a new preservation method is required to maintain the platelet function in ANH.

In our study, storage with highly gas permeable polyolefin maintained lower PCO_2_ and higher pH compared to PVN group (Table 2 and Fig. 2). Of ATP production by platelets, 85% is derived from aerobic metabolism^24,29^. In the 1980s, platelet concentrates were stored in PVC bags, but could not maintain the platelet function because of the accumulation of CO_2_ and lactate followed by a rapid decrease of pH^31^. Low pH generally causes platelet morphological change (below 6.7) and irreversible loss of viability (below 6.2)^30,32^. The whole blood stored in PVC bags in our study also showed a significant decrease in pH, but these declines were insufficient to cause the morphological changes. The polyolefin bags currently used for storage of platelet concentrates are 2.2 times more oxygen permeable and 3.6 times more CO_2_ permeable than PVC bags^22,33^. Use of high gas-permeable containers with gentle agitation ensures O_2_ and CO_2_ exchange between the storage medium and the atmosphere, and prevents local hypoxia resulting in lactate production^22,24^. This storage condition prevents PSLs and increases the ability to stop bleeding after transfusion^22,29^. However, it is unclear whether the gas permeability of the bag affected the aerobic metabolism of platelets in our results. Krause et al. showed that just limiting gas exchange in storage bag of platelet concentrates causes accumulation of PCO_2_, decreased pH, increased lactate, and increased P-selectin expression^34^. Similarly, the high gas permeability of the storage bag in our study may have led to higher aggregation rates and low P-selectin expression, although the underlying biochemical pathway is unknown. Mean platelet volume of platelets generally increases inversely related to pH, indicating a poor quality of product^35^, which did not change in this study. In contrast, there were no significant differences caused by agitation (Table 2 and Fig. 2, PVN–PVA, PON–POA). Thomas^24^ showed that interruption of agitation for several hours did not reduce platelet function. In addition to agitation, the platelet concentration and the surface area of the storage bag affect the oxygen partial pressure^33^. The effect of agitation on the platelet count and function may depend on the storage container, agitation speed and other conditions.

Preservative solution and temperature also influence PSLs. CPDA used in ANH is an anticoagulating solution suitable for long term storage of red blood cells in preoperative autologous blood donation^20^, but is not optimized for platelet storage. CPDA contains sodium citrate hydrate and dextrose, and the pH is 5.6–5.8. Several studies have reported that citric acid impairs platelet function even in short-time storage as practiced in ANH^12,36^. Hyperglycemia can induce hyperreactivity of platelets to high shear stress and increased P-selectin within 4 h^37,38^. For these reasons, CPDA is not a suitable solution for storage of platelets. The recommended temperature for platelet storage has changed over time. Platelet storage at 4 °C was performed in the 1970s, but 20–24 °C or room temperature is currently recommended based on reports that platelets stored below 20–24 °C rapidly change irreversibly and lose their viability after transfusion^24,32^. Room temperature storage is also recommended in ANH, but the temperature in the operating room during hypothermic CPB often falls below 20 °C, which may impair platelet function. However, some reports have indicated that cold-stored platelets contribute effectively to hemostasis^30,39^ and the US Food and Drug Administration recently approved cold-stored platelets in resuscitation of patients with active bleeding^40^. Therefore, we are planning the next study to clarify the appropriate preservative solutions and temperature for ANH, which provide better platelet function and reduce perioperative blood loss.

Red blood cell storage lesions are measured with indicators of hemolysis and the ability to deliver oxygen. There were no significant changes in hemoglobin concentration in all groups in this study. In general, several-hours whole blood storage at room temperature does not cause unacceptable injury to red blood cell functions^41^; 6-h whole blood storage at 20 °C with CPD decreases 2.3-DPG to 88%, which is acceptable^42^. Low pH suppresses the activity of glycolysis system, which in turn reduces 2,3-DPG required to supply oxygen to peripheral tissues and ATP production required to maintain erythrocyte morphology^43^, but 2,3-DPG and ATP levels recover in 7–72 h after transfusion^43^. High oxygen concentrations may confer oxidative stress, but the appropriate concentration range has not been determined^23,43^. Blood storage in polyolefin bags for several weeks are associated with greater red blood cells hemolysis compared to the storage in di-2-ethylhexil phthalate PVC bags^17^, but storage for several hours in our study had no effect. Mean corpuscular volume, a parameter of the erythrocyte morphology and storage lesions, tends to increase during storage^44,45^, but this also did not increase in our study. Red blood cells can be stored for a longer period than platelets, and the storage conditions such as higher PO_2_ for several hours may not affect their function^44,46^.

There are several limitations of this study. First, in order to reduce the burden on the volunteers, the amount of sample blood per bag was set at 20 mL, which is much less than the recommended capacity. Therefore, the surface area, internal pressure and effect of agitation in our storage bags might not be the same as those in clinical use. Storage with small amounts will allow easier agitation and gas exchange. Our results should be supplemented by full volume experiments in the future. Second, in addition to the material and gas permeability, the different surface structures and plasticizers between the two bags used may have influenced the results. Other products may have different effects on blood cells, because the structures of blood bag varies from manufacturer to manufacturer^16^. Third, ANH also improves CPB-induced coagulopathy^47^, but the effects of storage conditions of ANH on coagulation factors were not examined. Coagulation factors, even the most affected factor VIII, are maintained at more than 70%, which is sufficient for hemostasis, in 24-h whole blood storage at room temperature^48^. Global hemostasis assay such as thromboelastography or rotational thromboelastometry should be considered as an additional functional assay. Fourth, as this study was performed in vitro, the hemostatic effect and survival time and hemolysis in vivo of stored platelets could not be evaluated. Within these limitations, our results show that whole blood stored in a polyolefin bag for 6 h maintains significantly higher platelet function compared to the conventional method of storage in a PVC bag, while agitation had no effect on the results.

## Methods

**Figure 3:**
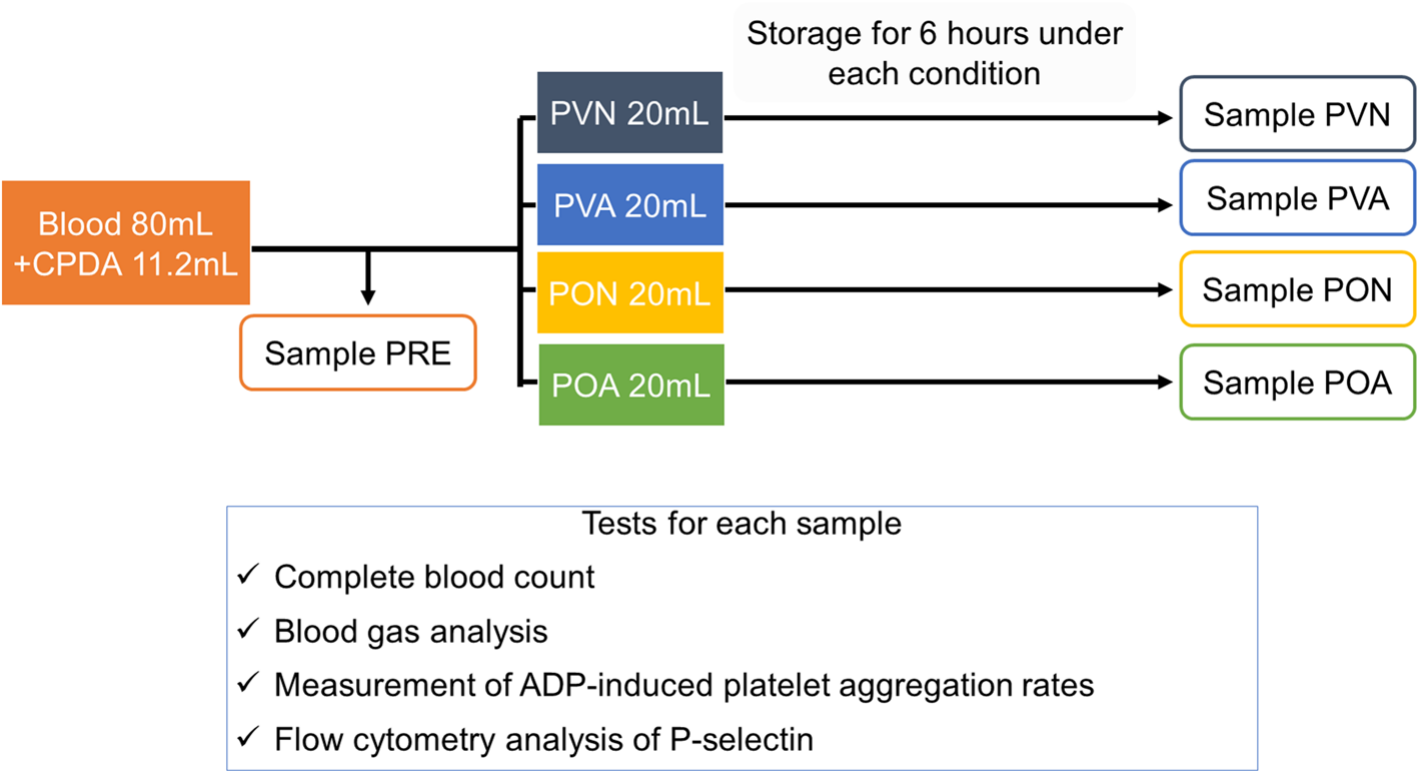
Flow chart of the study. Each venous blood sample from healthy volunteers was mixed with CPDA gently. After removed of a small amount of blood for tests (sample PRE), the rest of blood was divided for storage under each condition (PVN, PVA, PON, POA). After storage for 6 h under each condition, we tested them. This figure was drawn using Microsoft PowerPoint 2016 (Ver. 2103, https://www.microsoft.com/ja-jp/microsoft-365/powerpoint).

The study was approved by the ethics committee of Kyoto University Hospital (R0978-1) and carried out according to the guidelines of the Declaration of Helsinki. All methods were performed in accordance with the institutional guidelines and regulations. Prior written informed consent was obtained from subjects. A study flow chart is shown in Fig. 3.

### Storage groups

Four storage methods were examined: at rest in a PVC bag, as in conventional ANH (PVN); agitation in a PVC bag (PVA); at rest in a polyolefin bag (PON); and agitation in a polyolefin bag (POA). A Karmi CA (200 mL single, Kawasumi Laboratories, Inc., Tokyo, Japan) made of polyvinyl chloride including di-2-ethylhexyl phthalate plasticizer and containing CPDA solution was used as the PVC bag. A Kawasumi Separation bag PO (1000 mL single. Kawasumi Laboratories) made of polyolefin without any plasticizer and anticoagulant was used as the polyolefin bag. The O_2_ permeabilities of the PVC and polyolefin bags are 1.10 ± 0.04 and 2.37 ± 0.30 nmol/min/atm/cm^2^ and the CO_2_ permeabilities are 9.8 ± 0.5 and 35.43 ± 6.8 nmol/min/atm/cm^2^, respectively^33^. CPDA solution was removed from the PVC bag, and the volume of each PVC and polyolefin bag was adjusted to 20 mL by rolling the bag up and fixed with metal clips. A horizontal rotatory agitator (Labo Shaker BC-740, Bio Craft, Inc, Tokyo, Japan) was used for agitation at 60 rpm.

### Chemicals and drugs

ADP was purchased from Nacalai Tesque (Kyoto, Japan). Peridinin chlorophyll protein (PerCP)-labeled anti-CD61 antibody, and phycoerythrin (PE)-labeled anti-CD62P (P-selectin) antibody were obtained from Becton Dickinson (San Diego, CA, USA). The composition of CPDA removed from a PVC bag was citric acid hydrate 0.327 w/v%, sodium citrate hydrate 2.630 w/v%, monobasic sodium phosphate 0.251 w/v%, dextrose 2.900 w/v% and adenine 0.0275 w/v%. The pH was 5.6–5.8. All other chemicals were of analytical grade. It was confirmed that all buffers and solvents used for dilution had no effects on the results.

### Blood collection and storage

A sample of 80 mL of venous blood was collected by venipuncture of forearm veins from 6 healthy volunteers who had not taken any medication for at least two weeks before blood sampling. The blood was mixed with 11.2 mL of CPDA (8.14 v/v%; the percentage specified for use of PVC bags currently used in ANH) gently. After removal of 11 mL of blood for testing (sample PRE), the rest of the blood was divided into 20 mL volumes for each of the four groups. The blood was injected into 4 bags and extra air was removed. PVN and PON bags were set at rest, while PVA and POA bags were placed on a horizontal rotatory agitator at 60 rpm, with all 4 bags stored at 22 °C in an incubator box for 6 h. After storage, the blood was agitated gently and tested (samples PVN, PVA, PON, POA).

### Complete blood count and blood gas analysis

Each sample (PRE, PVN, PVA, PON, POA) was tested promptly after collection using the following methods. Complete blood counts and blood gas parameters were measured using an automated hematology analyzer (Celltac α Nihon Kohden, Tokyo, Japan) and an automated blood gas analyzer (RAPIDPoint 500 or RAPIDLab 1265 Siemens Healthineers, Munich, Germany).

### Measurement of ADP-induced platelet aggregation rates

Platelet-rich plasma (PRP) was prepared by centrifugation of a blood sample at 160*g* for 10 min at room temperature, followed by collection of the supernatant. The remaining lower portion was further centrifuged at 1600*g* for 15 min at room temperature and the clear supernatant was used as platelet-poor plasma (PPP). The platelet count was adjusted to 3 × 10^5^/μL by dilution with PPP (adjusted PRP). Aggregation induced by ADP was measured with a light transmission aggregometer (MCM Hema Tracer 212; MC Medical, Tokyo, Japan). Adjusted PRP (3 × 10^5^/μL, 200 μL) was pipetted into a cylindrical cuvette and incubated at 37 °C for 3 min, and then the adjusted PRP was stirred at 37 °C with a magnetic bar at 1000 rpm. A 10-μL volume of 200 μM ADP (final concentration: 9.5 μM) was added to the cylindrical cuvette and ADP-induced platelet aggregation rates was measured for 10 min as a change in light transmission from that of PPP, which was taken to be 100%.

### Flow cytometry analysis of P-selectin

Flow cytometry was performed as we have described previously^49,50^. Adjusted PRP was diluted tenfold with phosphate-buffered saline (PBS) (pH 7.42) containing 139 mM NaCl, 8.1 mM NaHPO_4_, 1.5 mM KH_2_PO_4_, and 2.7 mM KCl. Samples were fixed with ice-cold 1% formaldehyde for at least 60 min in a refrigerator and washed twice with ice-cold PBS by centrifugation at 1600*g* for 15 min at 4 °C. The pellet was suspended in 100 μL PBS at 4 °C. 20 μL of the suspension was coincubated with PerCP-labeled anti-CD61 antibody and PE-labeled anti-CD62P (P-selectin) antibody in a final volume of 100 μL adjusted with PBS for 60 min at room temperature in the dark. PE-labeled IgG was used to estimate nonspecific binding. The reaction was stopped by adding ice-cold PBS. Samples were analyzed using a fluorescence-activated cell sorting instrument (FACSCalibur Becton Dickinson, San Jose, CA, USA). For each sample, data from 10,000 platelets were collected. Platelets were identified by forward and side scatter intensity and by CD61 expression. P-selectin levels on activated platelet surface membranes were recorded as the mean fluorescent intensity (MFI) of PE.

### Statistical analysis

All data are expressed as a mean (standard deviation: SD) of 6 experiments. Group variances were tested by a Brown-Forsythe test and were statistically equal. All data were compared by one-way ANOVA, followed by a Dunnett test compared to PVN. All analyses were performed using JMP Pro 15.10 (https://www.jmp.com/ja_jp/software/predictive-analytics-software.html) (SAS Institute Inc., Cary, NC, USA) with *P* < 0.05 considered significant.
